# The rise of contract cheating during the COVID-19 pandemic: a qualitative study through the eyes of academics in Kuwait

**DOI:** 10.1186/s40468-021-00149-y

**Published:** 2021-12-02

**Authors:** Inan Deniz Erguvan

**Affiliations:** Gulf University for Science and Technology, West Mishref, Kuwait

**Keywords:** Contract cheating, Academic integrity, Faculty perspectives, Online learning, COVID-19 pandemic

## Abstract

Contract cheating has gone rampant in higher education recently. When institutions switched to online learning during the COVID-19 pandemic, the percentage of contract cheating students climbed to unprecedented levels. Essay mills saw the lack of face-to-face interaction and proctoring on campus as an opportunity and used aggressive marketing methods to attract students. This study asked the opinions of 20 faculty members working in the English departments of private higher education institutions in Kuwait regarding contract cheating through interviews. The data was analyzed with MAXQDA 2020. The findings show that all faculty members can recognize contract cheating easily. Most of them see contract cheating as a serious problem in the higher education system, a threat to the reliability of language assessment, triggered by laziness, the social pressure to graduate with a high GPA, and exacerbated by the cheating opportunities in online education. Academics have developed certain individual strategies in their courses to curb the number of contract cheating students; however, institutional measures differ, and in some, there are no measures or sanctions on contract cheating students.

## Introduction

In recent years, violations of academic integrity by students have increased and received attention from researchers, institutions, journalists, and policy-makers. While these violations vary widely, one emerging problem called ‘contract cheating’ has seen a global rise, across all disciplines. This sinister style of cheating has been aggravated “by the commodification of higher education and the increasingly popular sharing economy” (Williamson, 2019).

The phrase ‘contract cheating’ was first created by Clarke and Lancaster (2006). Contract cheating occurs when somebody other than the student does the assignment, passes it onto the student who turns it in to gain academic credit. Some argue that contract cheating should involve a monetary transaction between a student and a company (paper mill), whereas others define it as a student outsourcing his or her work, without necessarily having to pay anything for it (Eaton & Turner, 2020). It is worth mentioning that over the last decade, an industry, in which some companies or agencies, also known as paper mills, are paid to undertake this kind of academic work has emerged (The Quality Assurance Agency for Higher Education, 2020).

Contract cheating can be observed in any kind of written work, such as essay writing, science lab projects, computer-based projects and assignments, or any other technical work. Another point that needs clarifying is the difference between contract cheating and ghostwriting. Although two concepts are sometimes used interchangeably, the intention is not the same. For example, unless the celebrity is a well-known writer, it is presumed that he or she will be getting some assistance with their book. It may be acceptable to some extent to pay someone to ghostwrite a book; however, when contract cheating is involved in an assignment or a test, the instructor is deprived of a valuable tool to evaluate the student’s knowledge and score his or her performance reliably (Bretag, 2018).

Although students have been documented to pay third parties to carry out academic work in their name since the 1970s, with the advent of the Internet there has been a surge in contract cheating. Globally, universities are literally struggling to combat contract cheating. According to Lee (2019), in Australia, 16 universities were shocked by almost 1000 students utilizing a website to ghostwrite essays. The New York Times highlighted the rise of contract cheating in North America, in 2019. The Varsity Blues Scandal also clearly displayed that student were cheating to gain admissions into reputable universities and thus hiring others to complete assignments on their behalf (Lee, 2019). Bretag et al. (2019) stated that 5.8% of university students take part in one or more types of cheating; however, a high percentage of students participate in ‘sharing’ behaviors, such as buying, selling, or trading assignments for others. Studies from various countries have found the prevalence of contract cheating to range from 3.5% in Australia (Curtis & Clare, 2017) to 18.9% in Turkey (Eret & Ok, 2014). Also, a study in Czechia found 34% of students knew someone who got engaged in contract cheating, and 87% of students were aware of paper mills (Foltynek & Kralikova, 2018).

There are some strong indications that the potential for academic cheating has become even worse during the COVID-19 pandemic when universities all over the world had to shift to online learning. This shift has allowed more opportunities for students to complete assignments with online assistance; as a result, contract cheating has emerged as a real threat to academic integrity. Students believed that cheating in online exams was easier than the ones held in person; therefore, they tend to cheat more during online (King et al. 2009)

Many universities use software systems such as Turnitin, AntiPlag, TeSLA, and other software to detect plagiarism, and these systems are constantly improving (Pàmies et al. 2020; Jiffriya et al. 2013; Edwards et al. 2019). Nevertheless, none of these tools can reliably detect contract cheating. Some progress has been made in forensic linguistics to ensure academic integrity and punish academic misconduct (Peytcheva-Forsyth et al. 2019; Sousa-Silva, 2020), but technology is not developing fast enough to curtail all plagiarism practices, particularly, contract cheating.

While contract cheating seems to be triggered by an array of factors ranging from social, economic to cultural, and from educational, academic to personal (Awdry & Ives, 2020; Ali & Alhassan, 2021), one thing that most scholars would agree on is the ever-increasing visibility and aggressive marketing strategies of essay mills, particularly with the advent of social media. Essay mills have discovered innovative ways to satisfy students’ requirements and now they reach their potential customers through online advertising, emails, and phone calls. According to the e-book of contract cheating by the plagiarism detection software Turnitin (2021), essay mills have really made it easy for students to use their services by reaching students at the right time, offering a professional and allegedly legitimate experience, and following up students, i.e., their customers with perseverance.

Although contract cheating is mostly linked with essay mills, it surely does not apply exclusively to essay mills. According to Lancaster and Clarke (2016), students can use essay writing services, friends, family, and other students, and private tutors among many others. In fact, recent studies found that students are more likely to get help from people that they know (friends, parents), rather than from commercial sites and that money usually does not change hands with contract cheating (Armond & Varga, 2021; Turnitin, 2017). Indeed, 10.4% of students stated that they used a professional service, whereas 60.2% turned to a current or former student. From among the students who asked for help from other students, only 13.2% paid for this service (Turnitin, 2017). In many cases of contract cheating, friends or classmates informally exchange favors or just help each other out.

Contract cheating is a serious academic misconduct that threatens the academic integrity of the student’s grades and their qualifications. The consequences are not limited to individuals, as contract cheating also raises suspicion about all the degrees awarded by an institution. The effects of plagiarism and cheating continue even after formal education is completed (Williamson, 2019). Some studies demonstrated that undergraduate students, who engage in academic misconduct, are more likely to display inappropriate behaviors during their work life and there is a strong correlation between self-reported academic dishonesty and the level of corruption of a country (Guerrero-Dib et.al. 2020; Orosz et al. 2018). According to Bretag (2019), contract cheating is a threat to public safety as future doctors, engineers, and social workers who have outsourced their learning could pose a serious risk for the society. When researchers and scientists purchase their theses, publications, and qualifications, they will even endanger the credibility of science.

## Contract cheating in Kuwait

According to the Kuwaiti media, students engage in all types of academic dishonesty in Kuwaiti higher education institutes (AlSuwaileh et al. 2016). Indeed, the cases of cheating and plagiarism are generally reported in newspapers; however, there are very few empirical research studies on this issue. For example, a report revealed that one third of the university students admitted to buying papers, generally from shops around the campus which primarily seem to be providing printing and photocopying services (Al Jiyyar, 2017). The Kuwait Ministry of Commerce has closed such businesses from time to time due to complaints from the Ministry of Education, banning them from advertising research services, but particularly during the pandemic, most of these shops had to close and shifted their services to online platforms.

As these reports and studies (AlSuwaileh & AlRadaan, 2015; Al Jiyyar, 2017; Hamed & AlAhmad, 2018; Al Darwish & Sadeeqi, 2016) reveal, although academic misconduct is quite prevalent in Kuwait higher education institutes, they are not reported or sometimes go unnoticed. Students do not face consequences and professors are generally left to their own devices with plagiarizing and cheating students, as sometimes there are no academic integrity policies in place.

Although contract cheating is an increasingly challenging problem facing the higher education sector, literature review indicates research gaps deserving attention. Through a systematic literature review of 51 peer-reviewed articles on contract cheating in higher education, Ahsan et.al (2021) has found contract cheating research concentrates on only a few countries, such as Australia, UK, and Canada whereas the USA, China, India, and other emerging countries remain under-researched. Moreover, various contextual aspects which may influence contract cheating such as society, culture, and religion have also not been adequately explored. Another topic that has not been researched yet is contract cheating during and in the post COVID-19 era.

Thus, this paper will investigate contract cheating in private higher education institutes in Kuwait, an under-researched country in this sense. The existing literature on contract cheating in Kuwait is indeed scarce and the very few studies found mostly focused on students. The faculty members’ viewpoints regarding how COVID-19 has influenced contract cheating practices of students will also be analyzed in the study, a research gap indicated by Ahsan, et.al (2021).

This research is unique in a sense that it will be the first one investigating contract cheating to gather empirical evidence in a Kuwaiti context through the eyes of the faculty members working in private higher education institutes. The use of commercial contract cheating services is a borderless phenomenon; the student, their university, and the writer and company could all be in different countries. Therefore, we can assume that country-specific analysis will contribute to the higher education literature and help higher education sector to combat this form of academic misconduct.

The key questions in this study will be

1. What is the overall awareness level of faculty members in recognizing contract cheating?
2. How has online education during the pandemic affected contract cheating?
3. What do faculty members suggest as a solution to combat contract cheating?

## Methodology

The research design in this study could be described as exploratory qualitative, as participants’ perspectives were obtained through semi-structured interviews. The questions in the interview were developed to gain participants’ perceptions of contract cheating in the courses that they teach within their institutes. The questions were adapted from similar studies conducted by Ali and Alhassan (2021) who conducted a qualitative study among higher education institutes in Oman, a culturally similar country to Kuwait; and Awdry and Newton (2019) who surveyed staff views in Australian and British universities via a questionnaire. This study has expanded on these previous studies by including questions assessing faculty members’ viewpoints regarding the impact of COVID-19 on contract cheating.

### Research population

The population of the research is 20 faculty members who were employed in four different private higher education institutions in Kuwait, teaching English language, literature, or writing skills, during the 2020–2021 academic year. The researcher used purposive sampling to access faculty members who would most likely have the experience to provide quality information and valuable insights on the research topic. According to Layder (2013), in purposive sampling the sample is ‘handpicked’ for the research, where the researcher already knows something about the specific people or events and deliberately selects them because they are likely to produce the most valuable data. This type of sampling aimed to ensure that the sample is as diverse as possible to be able to identify a full range of perspectives that are associated with contract cheating within the Kuwaiti higher education context.

The sample was selected according to the following criteria: fulltime faculty members working in various private higher education institutes in Kuwait during the pandemic who agreed to be interviewed and recorded in an online interview and signed the consent form. The participants in this study represent different disciplines, institutes, and national and cultural backgrounds.

### Data collection

The faculty members were interviewed on an online platform due to the restrictions of the COVID-19 pandemic. Interviews offer an advantage over surveys as researchers can get more details on vague answers. According to Brown (2001), interviews have a high return rate and fewer incomplete answers.

For the reliability and validity of the interviews, the researcher conducted some pilot interviews with some faculty who are not in the sample to check the understandability of the questions. After the interview, the recorded voice file and the written interview text were sent to each interviewee to obtain their approval to avoid any misunderstandings.

Due to the interactive nature of the interview and the various biases and limits that may impact human decision-making, the interviewer did not deviate from the interview questions and kept a neutral body language with all interviewees. After the interview, the recorded voice file and the written interview text were sent to each interviewee to obtain their approval to avoid any misunderstandings.

### Data analysis

Data analysis in qualitative research includes preparing and organizing the data, coding it, and bringing the codes together and reducing them to themes, then presenting the data in the form of figures, tables, or a discussion (Creswell, 2018). The data obtained in this research were examined through thematic and content analysis methods.

Thematic analysis is a method used to identify, analyze, and report the themes in the obtained data, and it enables the data to be organized and described in the smallest dimensions (Boyatzis, 1998; Braun & Clarke, 2019). Content analysis is the systematic, objective and, if possible, quantitative analysis of the content of various documents (Bilgin, 2006). The main purpose of content analysis is to reach concepts and relationships that will help explain the collected data. While the introductory findings of the participants are evaluated with thematic analysis, the content of the participants’ opinions is systematically examined with content analysis (Karatas, 2015).

For data analysis, the audio recordings obtained from the interviews were transcribed. To protect the anonymity and confidentiality of the participants, each interviewee was given a code (P1 to P20). The institution names were also removed from the transcribed interviews.

Written interview data were transferred to the MAXQDA 2020 program. The MAXQDA program, uses visual analysis tools extensively, and can be used in mixed research methods in addition to basic statistical analyses, provides a more systematic analysis of data compared to hand-held analysis (Kuckartz & Rädiker, 2019). An inductive approach has been adopted in the analysis of the data transferred to the MAXQDA 2020 program. The data was read repeatedly, and the first codes were generated. Codes related to each other were grouped under the same themes and named relevantly. Afterwards, the themes obtained were explained in detail. Finally, the researcher interpreted the findings and supported them with various visuals.

## Results

### Research question 1

The first research question of the study was geared towards analyzing the awareness level of faculty members in terms of recognition and detection of contract cheating. The perception of contract cheating theme has been examined under 3 different categories. These are awareness of contract cheating, telltale signs, and the most common cheating strategy.

#### Awareness of contract cheating

Awareness of contract cheating category was defined with three different codes. These are: being aware of contract cheating, receiving such assignments, aware but not being able to prove cheating. Most participants expressed that they are aware of contract cheating taking place around them, and in many ways:

*“I was surprised to see how common it is in Kuwait. Last year, some of them even tried to contact me on Instagram, obviously having no idea who I am and where I work. They were the ghostwriters themselves, thinking that I was a student" (P7)*

*"Yes. I'm very aware with that. And this thing became evident, especially now with our online teaching. You can see a clear distinction between the linguistic fingerprint of the students and of the one answering the questions.” (P8)*

In the same category, participants expressed they receive such cheated or plagiarized work from their students:

*“I have had many students, even before this online environment. After I pressed them, this is plagiarized, you could not have written this yourself, they would, tell me I've had my friend, family member, sister, whoever, helped me.” (P4)*

*“Yes, I have received some assignments. I suspected that they are ghost written assignments because, the level of assignments was very high for those students, for example, their English is not very good, but the assignment language was very high.” (P14)*

Some participants mentioned that they have a hunch that students cheat, but they cannot always prove it: *“I don't have a lot of evidence. This is the problem, because I know that it doesn't matter whether they turned the camera on, there could always be somebody there doing it for them” (P5)*

#### Telltale signs

**Fig. 1:**
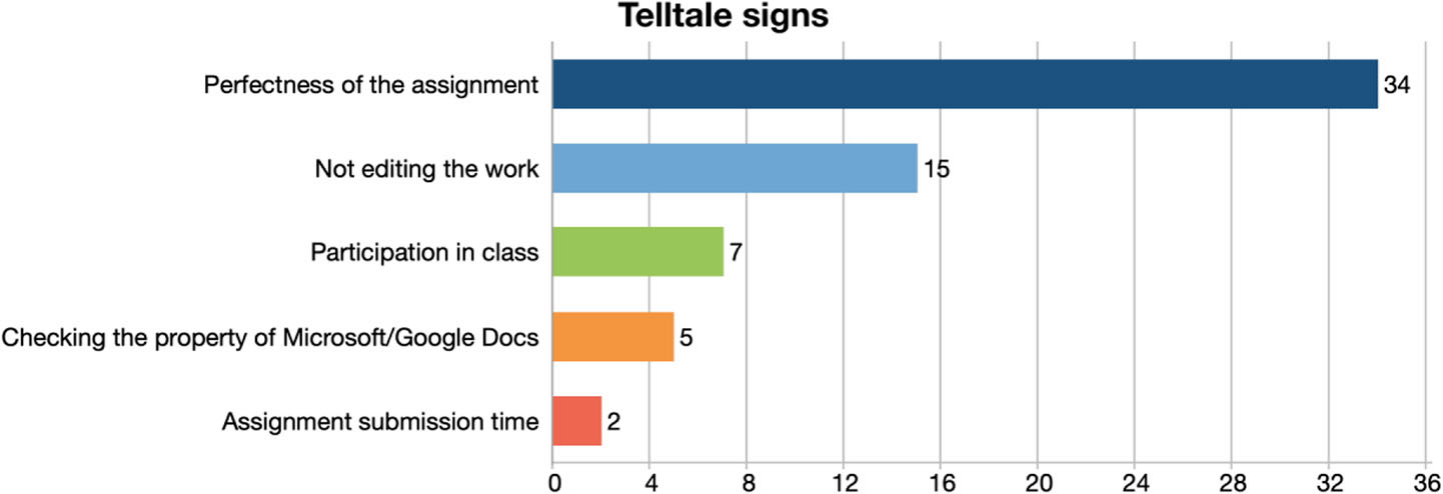
Telltale signs of contract cheated assignments

This category was defined with four different codes: perfectness of the assignment, not editing the work, checking the property of Microsoft/Google Docs, and assignment submission time, as displayed in Fig. 1.

Participants mentioned that contract cheating students submit assignments that are grammatically flawless and much better than their real level:

*“But I look up the GPA, there’s somebody who has a very low GPA, he's really struggling and then he has an essay, and you would like to publish it in the newspaper because it’s so good… well then it's clear.” (P1)*

*“Well, one would be a flawless essay written by a student who can barely speak English and we still know our students, even though we teach online, they speak in class, or they send us messages that are sometimes ungrammatical. So, we know students who have given us work, that's produced by someone else.” (P11)*

Another code participants mentioned was that contract cheating students do not modify their submission according to the specific instructions of the given assignment. The work taken from the ghostwriter generally does not follow the template that the instructor has shown in class:

*“Some of the essays are received from my students were written in a totally different manner, not necessarily incorrect but just different than the one I told them in terms of the components of the essay, the order of the ideas to follow… So, in that sense, it is easily noticeable.” (P7)*

Unusual file properties after checking Microsoft/Google Docs make participants suspect that somebody else has contributed to the assignment:

*“I have instances where I can check the properties Microsoft submission or PowerPoint, and you can tell who forgot to remove their name from the properties. And tell, even it's a certain paper mill. You can look it up on Google and find out how much they charge.” (P2)*

Assignment submission time, whether it was too late or early was seen as a red flag by some participants.

*“Some of my students submit work 20 minutes late. We have some timed assignments; they have to write it and finish in class on time. 60 minutes is still long enough, but if they are communicating with their buddy, … the communication fails, so they're desperate to submit their work 10 minutes 20 minutes later, depending on the source.” (P4)*

*“Because we use Pearson, students who cheat start the quiz later, they don't start on time, 25-30 minutes later and then they finish in seven minutes instead of 45 minutes, so it's just not possible to finish in seven minutes or 20 minutes and submit something perfect.” (P20)*

#### Most common cheating strategy

The most common cheating strategy category was defined with two different codes: paper mills and friends and family. Participants mentioned that the most common cheating strategy that they encounter is paper mills:

*“With the incidents that I found, I found 20% of them to have family members, that have majored in either English or good at English to write their essays. But the majority 80% is fee-based.” (P8)*

*“Students are now familiar with Turnitin and that the old submissions will be immediately detected; therefore, they are now avoiding those types of help. So, ghostwriting, I mean cheating agencies, yes, this is what I have seen in the recent years.” (P12)*

*“I think before COVID, it was primarily friends, family members, relatives, other classmates that were helping them. And it wasn't necessarily a financial transaction.” (P6)*

### Research question 2

This question was designed to analyze the perceptions of academics regarding the rise of contract cheating during the pandemic. Most participants perceived the numbers to have risen:

*“I observed a massive rise in academic dishonesty during online teaching, across the board, in contract cheating, as well. I think that is just off the chart cheating in everything. I have very low confidence in the integrity of the grades that are currently being produced.” (P3)*

*“Online has definitely increased the amount of a quiz cheating. When we were on campus if I suspected somebody, I'd bring them to my office and then, interrogate them and they’d be a little intimidated. So, probably they would admit. How can I interrogate the way that I was doing the class in my office? I cannot.” (P5)*

Some participants mentioned that contract cheating during the pandemic has not risen, or they were unsure if the pandemic has made any impact on the number of cases:

*“I'm not sure because I've always been able to detect contract cheating. I know their strategies have shifted because things are online. So perhaps other instructors are finding an increase, but I think that the increase has always been there, in the last several years.” (P2)*

When asked how often they are submitted contract cheated assignments, most participants expressed approximately 0–25% students resort to contract cheating in their courses. Some quotations regarding different perspectives of percentages are given below:

*“It depends on each given semester. But I have a class of 28 you might find like five of them that do it. In a class, you might find like three or four and I have one smaller section of 14, you might find like 2.” (P2)*

*“I would say it could be anything close to 40-50%. It's happening a lot.” (P11)*

*“The thing is before the pandemic, they weren't able to cheat during the actual midterms on the finals, at least not to this point, but now after the pandemic, I can say this number has gone up to probably 98 or 99%” (P17)*

**Fig. 2:**
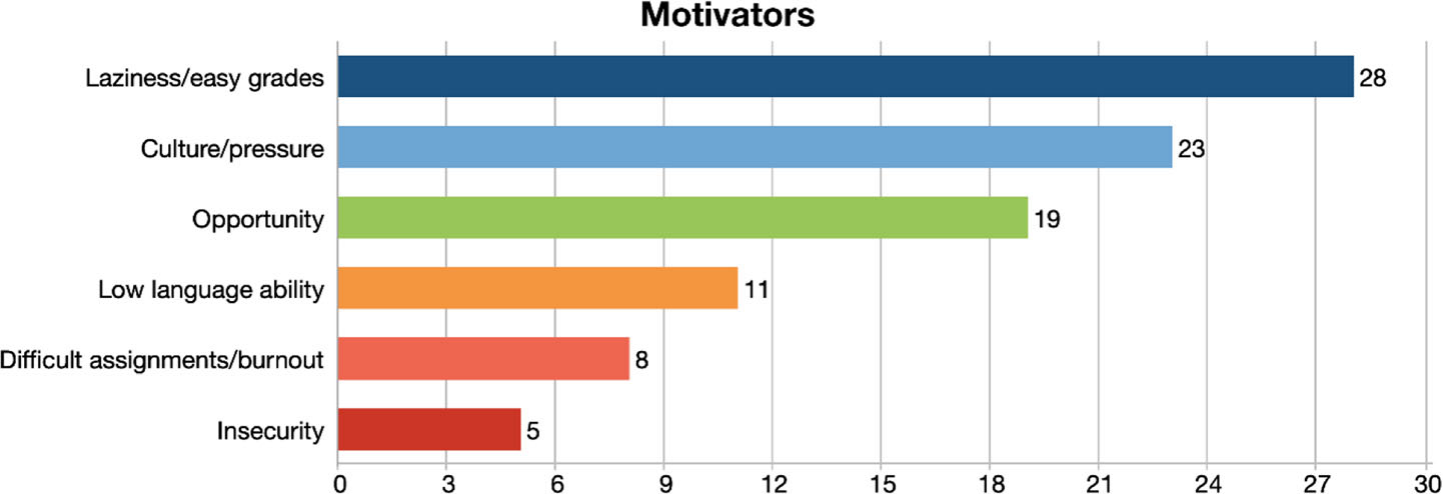
Motivators for contract cheating and frequencies

The faculty members were also asked what they consider the major motivators for the rise of contract cheating during the pandemic. The responses to this question in terms of codes could be seen in Fig. 2

Participants mentioned that student’s reasons to contract cheat are their laziness to do the necessary work and wish for easy access to good grades. The participants said the following:

*“I believe the first reason is the reluctance to make an effort, going to college or studying in a certain program.” (P7)*

*“Lack of motivation and lack of willingness to learn because it's a learning process, that's not something that some people are interested in” (P13)*

The second most frequently mentioned code was the culture and the social pressure to be university graduate in Kuwait:

*“This is a very common thing in our society unfortunately. They're very lazy, but they want the grade because it's for their reputation and for the society.” (P15)*

Some participants also brought up opportunity as a motivator. Opportunity in this context involves the absence of proctoring; having money to buy the work; and easy access to such services.

*“The opportunity is there constantly. Once these essay mills realize that this guy's a student at university, through social media, friend of a friend, they contact them. So, the opportunity presents itself. And I think in some places, students feel they are a fool not to take advantage of it.” (P9)*

*“They have money, and they know that they can hire someone easily. So sometimes I think a group of students are sharing the money. So, it is not a costing a lot for them.” (P14)*

Participant 18, linked the rise of contract cheating to COVID burnout:

*“Maybe one of the motives that's inspiring people is burnout. And everyone has a bit of COVID burnout. They're at home isolated, I think that burnout might be part of the incentive that people find it harder to get motivated.” (P18)*

#### Research question 3

**Fig. 3:**
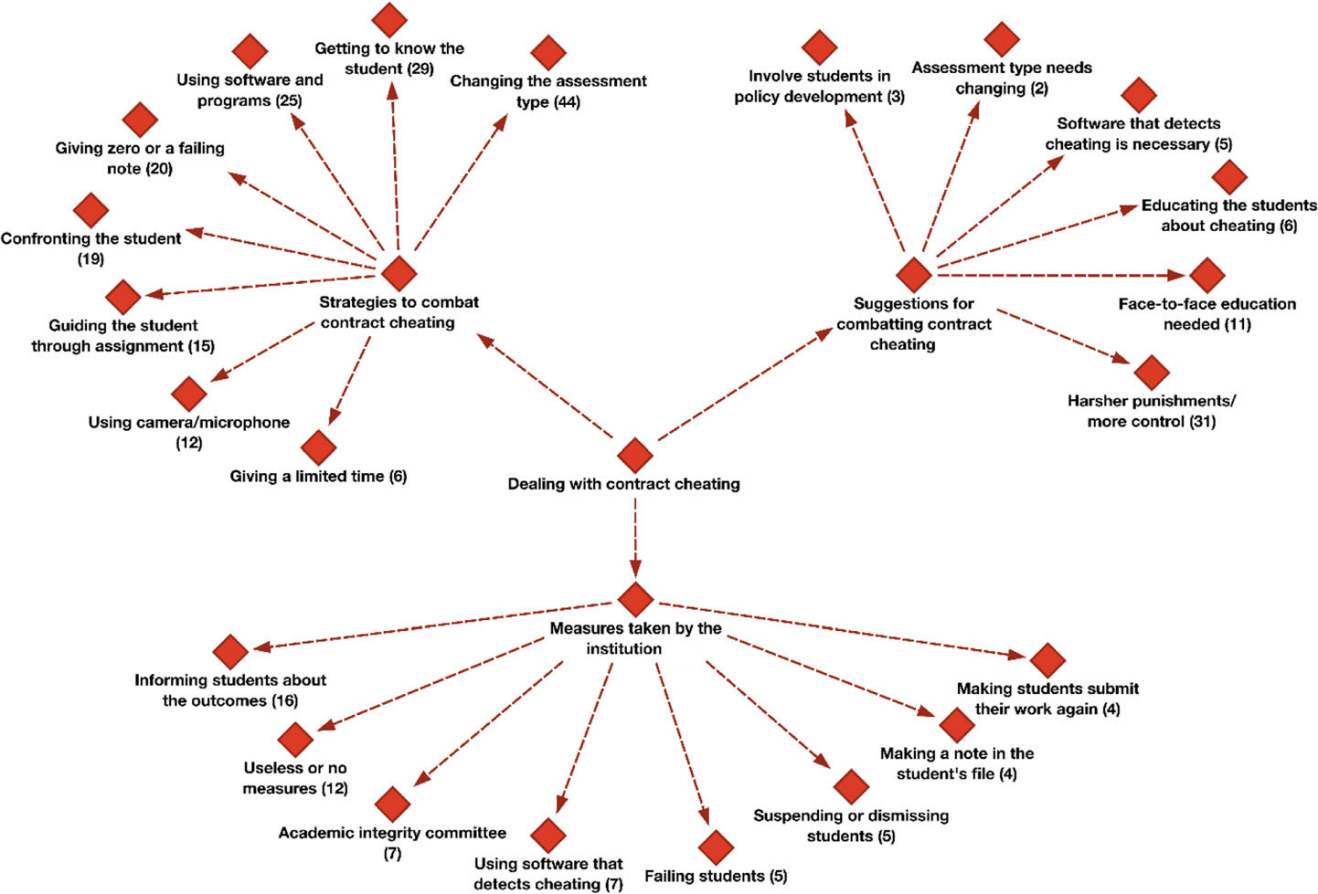
Dealing with contract cheating hierarchical code-subcodes model and frequencies

When asked about the solutions the faculty members could suggest helping eliminate the problem of contract cheating, their responses revealed 3 different categories: personal strategies to combat cheating, institutional measures taken to combat contract cheating, general suggestions for combatting contract cheating, as displayed in Fig. 3.

#### Strategies to combat contract cheating

**Fig. 4:**
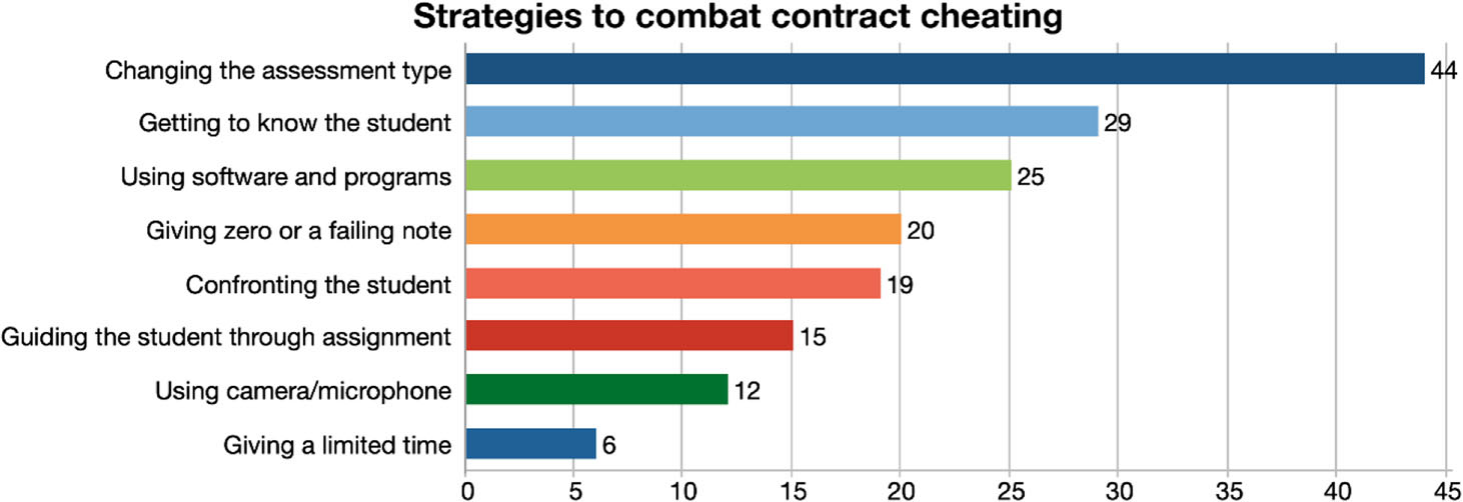
Strategies to combat contract cheating

Eight different strategies that faculty members have adopted are shown in Fig. 4.

Changing the assessment type was the most adopted strategy, particularly during the pandemic. Participants mentioned that they change both the type of assignments and the questions that they ask in their exams or quizzes:

*“I've reduced the amount of anything in my course that is written…So now, everything in my class is pretty much presented orally.” (P2)*

*“We revised the type of assignments that we had students do. We accomplish the curriculum goals in a different way, using a different kind of assignment. We just worked around it by using a different activity.” (P6)*

The next most frequently mentioned strategy was getting to know the student, trying to find out the true level of the student throughout the course:

*“Before giving an assignment, I would usually ask these students to do specific tasks, a writing assignment, an essay… So, when I receive their essays, I have their linguistic fingerprint. In case you suspect contract cheating, you go back to previous essays, you will see a discrepancy in the quality of the way it was written.” (P8)*

Using certain software to detect and prevent the contract cheating in exams or assignments is also a commonly used strategy:

*“…during the assessments we used an app called lockdown browser which disables access to all the websites and apps, other than the one used for the assessment.” (P7)*

Some participants confront the student, and give their cheating students a 0/F or low grade that is enough to fail that student in that course:

*“Sometimes I've caught people and said, “The writing style in this assignment is so different from the other things that you've submitted.” then they'll say, well, my sister helped me or, or I had a friend or something like that…” (P18)*

*“I would just give them a zero and that will drop their grade, which is a huge punishment. Cause it drops the grade big time, 20% or 25%, without a possibility of doing a make-up...” (P13)*

A few participants mentioned that if faculty members are involved in the whole process and guiding the student during that time, cheating will be much harder, and students will not resort to contract cheating:

*“I never give them an assignment and say, bring me a 30-page paper, with 50 references. I tell them, I want you to know exactly what you're doing... then I want to know your methodology. How do you want to research these questions? … show me your interview questions. Who are you going to interview exactly and why? Like baby steps...” (P13)*

As well as using camera and microphone in online teaching, some participants mentioned that they prevent students cheat by giving them as little time as they can only do the exam or assignment:

*“In my opinion if you want honest answers, create a huge question bank, randomize it and the key is, time pressure. Otherwise, if you give them enough time then you'll get perfect answers across the board because it's all debated in the WhatsApp group.” (P3)*

#### Measures taken by the institution

**Fig. 5:**
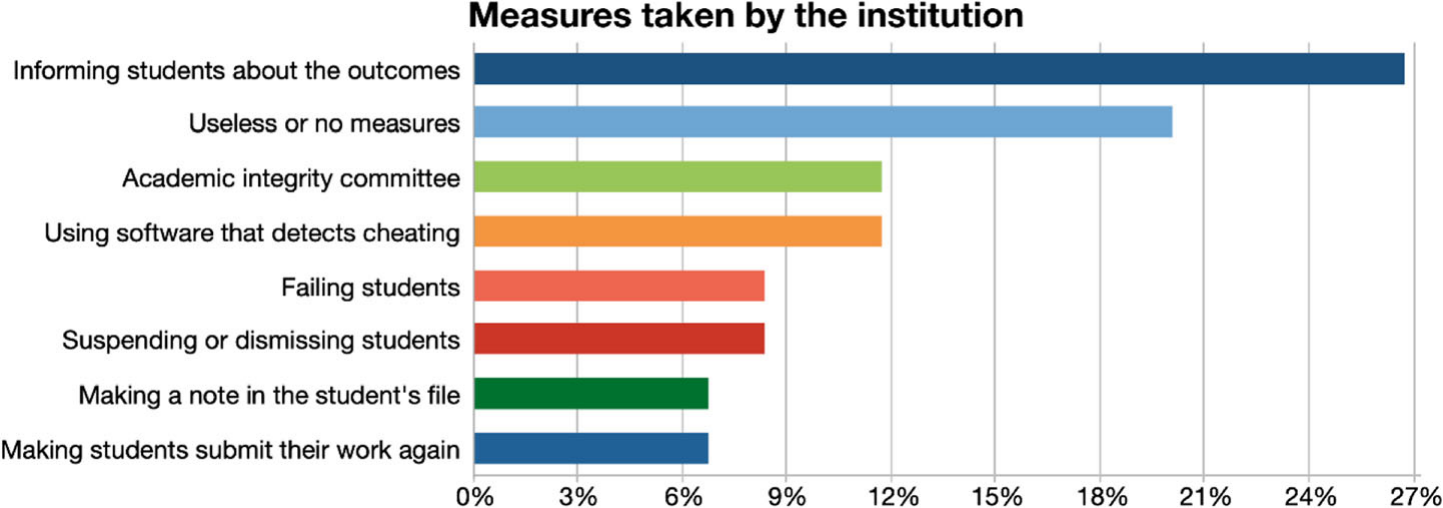
Measures taken by the institution

The measures the institutions are taking to curb contract cheating could be classified in 8 different codes, as could be seen in Fig. 5.

The most popular measure taken by institutions is informing students about the outcomes, through syllabus, integrity statements or in classes, verbally:

*“Every university calls it different. I remember in Iowa, we used to have those things that students have to sign, and they’re very well aware, if they are caught cheating, this is not going to be your decision anymore. This is a contract. I'm sorry but you have to be directly sent to the head of department to deal with you, and the head of department should only tell them, you signed here.” (P5)*

The next code mentioned by participants is there were no institutional measures. Some participants mentioned some institutions take no action against cheating:

*“We haven't talked about it in our department. Until recently I think they were not aware of it. They always say Turnitin, and that's good and then you see that it doesn't control the internet. Contract cheating is a completely different matter and, no, we haven't talked about it” (P1)*

Some institutions have an academic integrity committee to deal with contract cheating:

*“We have a committee, a great appeal committee or a committee to look at this. They are more willing to step up because they don't have to deal with the student. It's more like a collective issue, it’s not a one-on-one battle with them.” (P6)*

*“When we have the anti-cheating rules and how I know that if the case gets to the academic integrity committee, ... I was part of that committee, and the consequences were very dire…very severe.” (P8)*

Participants expressed that if the student is caught cheating, they will fail that course, as an institutional policy. More severe measures would be suspending the student from the university, and/ or making a note in the student’s file:

*“Cheating in any assignment is considered as serious academic misconduct, any work that has been copied plagiarized or completed by someone other than the submitting student will get a zero.” (P14)*

*“There were several instances of ghost writing, the students were caught and kicked out of university for a semester” (P6)*

Some institutes were quite flexible with this academic misconduct and participants said they give the student a second chance to submit their work again, with a small penalty:

*“We give them only a two-day window. So, if they resubmitted the work within the first 24 hours after we emailed them, they lose 40% of the grade.” (P17)*

#### Suggestions for combatting contract cheating

**Fig. 6:**
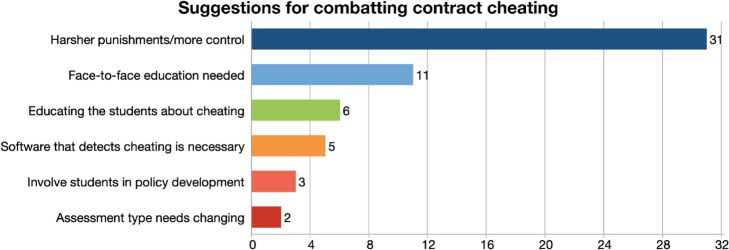
Suggestions for combatting contract cheating

Suggestions for combatting contract cheating category were defined with 6 different codes, as shown in Fig. 6.

Participants mentioned that the institution should take stricter, deterrent punishments and have more control over students to prevent cheating:

*“The only idea that we had that would probably make them not do that is to implement some severe punishments.” (P5)*

*“…to have clear and severe consequences for cheating. They should make it clear that if a student receives an F in any course, due to plagiarism his case should be reviewed by a panel, and he should be taken off the government scholarship.” (P8)*

Another suggestion mentioned was the need for face-to-face education. Participants expressed there is more control over students in face-to-face education, so cheating takes place less often:

*“The moment they come to campus, there is no way to cheat. Even if they continue to take the exam on their computer as long as they are in class with their computer, and the phones are not with them, they are under control… if we can bring them here only for the final exam, this is good enough to control everything.” (P20)*

Participants also expressed students need to be educated about cheating at young ages:

*“Maybe it takes some time, but so they can attend plagiarism seminars. We can start this from lower grades from schools, from middle school, maybe a teacher explains why this is wrong. So maybe culture can change in maybe next 10 years... So, we have to start this during childhood, not when they are adults.” (P14)*

*“You need positive encouragement, you need training, for students as well as for faculty… training for students, at the foundation level and the early years especially, focusing on the importance of learning, how to learn as the main benefit of education, rather than just getting a degree that gets you a job.” (P18)*

Some participants brought up the need for some software that detects cheating. Participants mentioned that most institutions use certain programs and browsers that detect cheating:

*“We need to use a lockdown browser or some serious software. I mean, we're using freeware now, safe exam browser doesn't cost anything.” (P6)*

A few participants mentioned that students should be integrated in the cheating policy development process:

*“In order to raise students’ awareness of academic dishonesty policy, higher educational institutions should involve students in this process, because when students are involved, they can also suggest measures to prevent the ghostwriting or any form of plagiarism. Students can share their rules and the policy brought by the university, with their peers, with other students.” (P12)*

Changing the assessment type was also brought up as a suggestion to combat contract cheating:

*“I think amending assessment is one of them. Maybe we need to get away from the traditional kind of idea of ‘Here is the essay. Here are the essay questions. And this is the deadline…’ because that just gives them a window of opportunity to cheat, to order their essays.” (P11)*

## Discussion

The findings regarding the first theme of this study, which is perceiving what contract cheating is, show that faculty members are aware of contract cheating, all have received contract cheated assignments or know a colleague who has received such an assignment. They can detect an assignment or an exam that has been produced by somebody else due to the perfectness of the work produced, discrepancy between the level of the submitted work and students’ level displayed in class or in written communication, and by looking at technical details such as submission time and file properties in Google docs and Microsoft word.

These findings are in line with other studies conducted pre and post pandemic, in other countries. In a study conducted among Omani university instructors (Ali & Alhassan, 2021), participants expressed that contract cheating is a serious and difficult to detect form of plagiarism which can threaten academic integrity. A survey of eight Australian universities found the most common signals that prompt faculty’s suspicions are the mismatch of their knowledge of students’ academic and linguistic abilities and the quality of student work (Harper et al. 2019). Indeed, although some programs claim to be able to identify authors by their style, a ghostwritten work is probably original and will not be detected by software alone. Singh and Remenyi (2016) suggest that contract cheating can be detected only if the evaluator is personally acquainted with the student’s level of subject knowledge and his or her natural writing style.

The participants in our study linked contract cheating to motivators such as laziness, desire to get easy grades, the ease of access to contract cheating services and the cultural and social pressure on the youth to finish university with a high GPA to increase their chances of getting a job in the public sector. The reasons to resort to contract cheating in the literature have been listed as the perception that there are lots of opportunities to cheat, increased accessibility contract cheating services, students’ (mis)perception that cheating is easy, challenging workloads, and lack of motivation and personal factors such as gender, personality, age, and grade average point (Bretag et al. 2019; Lines, 2016; Gullifer & Tyson, 2010). Awdry and Ives (2020) found personal factors, discipline, and country do not predict contract cheating, and likewise the participants in our study have not linked personal factors to the rising numbers of contract cheating. As some participants expressed, stress factor during the pandemic has motivated some students to take the easy way out. Studies from Bangladesh (Khan, et al. 2021), Hong Kong (Mok, et al. 2021), and Vietnam (Tran, et al. 2021) also found that students mentally and psychologically struggled during the pandemic, and some showed signs of depression; they did not find online learning satisfying; they lacked the computer skills and sometimes the equipment to complete online assignments.

As for our prevalence finding, although participants mostly agreed that 25% of their students are contract cheating, some expressed a much higher percentage. Most of them believe the number has jumped during the pandemic, due to the opportunities provided by online learning platforms. Lancaster and Cotarlan (2021) compared pre- and post-pandemic numbers of student applications to a file sharing platform and saw the numbers up by 196.25%. This big jump coincides with the time when many courses moved to be delivered and assessed online. Sarah Eaton (2020) said the increase in contract cheating has gone from about 40% to over 200%, based on reports published by schools across the country, in Canada. Similarly, according to Keate (2021), in the University of Waterloo in Canada cheating rose by 146% in August 2020, in the University of Calgary by 269%. compared to the previous year. The Quality Assurance Agency in the UK (2020) found out during the COVID-19 around 900 essay mills were providing ghost-written essays.

Our findings indicate that academics are convinced that during the pandemic, paper mills, rather than family members, have been behind the rising contract cheating cases. Participants have expressed their opinion that online learning has provided more opportunities for students to contract cheat and has tempted more students to get easy grades. Eaton (2020) suggests that students who previously did not give in to such companies “have found themselves bombarded with offers of help on social media from predatory commercial enterprises wanting to make the most of a stressful situation” (p. 83). This study supports our finding which basically suggests there are more opportunities for students to contract cheat in online learning. Eaton (2020) lists the reasons of increased contract cheating during the pandemic as faculty members not adapting their assessment to e-learning, lack of awareness about students’ file-sharing platforms, which include exam questions and answers, students’ increased stress level during the pandemic which may lead to academic misconduct, and aggressive marketing strategies of commercial contract cheating companies during the pandemic.

Our findings also focused on individual strategies, suggestions, and institutional measures to prevent contract cheating. Most participants agreed that informing the students of the consequences of cheating, changing the assessment type are some of the successful strategies in this regard. Getting to know the students better, assessing their classroom participation, using various software to monitor their activities during an exam were all adopted strategies by the participants in our study. The importance of making students aware of the consequences, was emphasized by some faculty in our study and in University of California (Reddin, 2021), faculty members put together a strongly worded honor statement and asked all students to sign it to be able to take the exam. By this way, students were given a clear message that if they violated the honor code, they could get dismissed from the university. This honor statement became successful and reduced the number of contract cheaters in the university.

Awareness and detection of contract cheating by academics alone is not enough to ensure that universities effectively deal with the problem. Some measures should be implemented by institutions, policies and processes should be clearly communicated to students to maintain academic integrity. Some participants have expressed that the institutions they work for have taken no measures to combat contract cheating or they are not applying them consistently. Similarly, Harper et al. (2019) found that students are prone to rationalizing cheating when there is a perceived lack of care or interest from academic staff or the university. A study conducted on Iranian ELT students (Ahmadi, 2014) also revealed that having lenient professors is a major reason for engaging in plagiarism. When professors do not show enough care in dealing with plagiarism, students consider plagiarism an easy task.

Exams are a significant element of university level language assessment, and they need to adapt to an online environment to ensure integrity. Most participants in our study pointed the significance of changing assessment types. Bretag et al. (2020) asserts that while there no assessment type was perceived to be immune to outsourcing, assessments that are least likely to be outsourced were ‘reflection on practicum’, ‘in-class task’, ‘personalized and unique’ assessments, and viva voces. Adjusting exams to online environment and using oral assessments is also suggested by Hillier (2020). Williamson (2019) maintains that designing assessment, which is meaningful, reasonable, timely, and closely linked to learning outcomes is the institution’s responsibility. Assessment can be designed to both reduce the chances of cheating and increase the chances for students to demonstrate their understanding: an oral examination of written assignments would help.

Use of software or turning cameras on was also brought up by some faculty members to ensure academic integrity in exams; however, some of these strategies are controversial. The financial cost of such software may be overwhelming (Hillier, 2020), also online proctoring services providing security measures such as biometric data, eye movement, and keystroke tracking, may be violating privacy (Hill et al. 2021). With the use of such services, the good will of some students “who may feel the surveillance is so intrusive it breaches their basic rights” may be lost (Hill et al. 2021). Thus, as some participants in our study stated, going back to face-to-face learning, even only for final exams would ensure exam security. Hiring large centers to ensure social distancing or conducting exams outdoors with an increased number of invigilators is a viable solution (Harper et al. 2019).

## Conclusion and recommendations

Overall, the findings of the study reveal that faculty members have a high level of awareness of contract cheating. They believe online learning has created more opportunities to contract cheat and thus, a minimum of one quarter of their students are resorting to essay mills in the online learning environment. They also believe some cultural habits such as a desire to have everything easy, laziness, and social pressure to graduate with a university degree are acting as incentives to resort to contract cheating. Faculty members did not show much difference in their recognition of contract cheating regarding the institution they work for, although measures adopted by the institutions were different. It was obvious that there was a lack of consistency of measures taken at the institutional level, with faculty members developing their own strategies, and institutional procedures initiated in only some cases. Some private universities did not have any strategies or measures to combat this misconduct, whereas some had honor codes, academic integrity committees and various software tools to curb contract cheating. Faculty members think going back to face-to-face teaching will alleviate some of the problems they are confronting now, but even if they must continue online for some more time, changing the assessment types and using more interactive approaches with their students is the best weapon they will be using to deal with contract cheating.

Faculty members certainly differ in their experiences of academic integrity in their relevant institutes which result in their adoption of different approaches on how to sanction contract cheating. It was not possible from this data to determine the reasons for these differences among academics, but it will be significant to explore and address these differing perceptions in further studies.

While the use anti-plagiarism software and vigilant proctoring was widely accepted, some inconsistent policies or not so user-friendly properties of these tools were also acknowledged. Institutions should explore these technological tools carefully and invest some money on installing them and training their staff and students to ensure academic integrity across all universities.

Also, while trying to ensure academic integrity and developing policies, institutions need to recognize that the faculty perspectives may not be shared by students. High school and college students should be trained about ethical values, the significance of academic integrity in university, and its consequences for their future careers. This should also entail consultation with students about institutional policies and their implementation. In recognizing academic integrity as a shared cultural issue, institutions and academics will need to pay more attention to students’ perspectives in this area.

Universities must ensure that their assessment processes are reliable and transparent, and that the value of qualifications awarded to students is in line with standards. Unfortunately, contract cheating services, and the students resorting to this misconduct constitute a risk to achieving this. Contract cheating is not just the responsibility of individual students, academics, or institutions, but it is a universal issue which government agencies, regulatory authorities, and leaders in higher education should be involved in. Higher education institutes, policy, and decision-makers should acknowledge the problem of contract cheating and along with student bodies, faculty members, and other universities, should work towards increasing academic integrity across the higher education institutes in the country.

## Limitations

There are some limitations to the methodology used in this article. The sample size was modest but provided rich qualitative data. We used a convenience sample, i.e., faculty members and institutions were chosen for this study because they were accessible to the researcher. Thus, we cannot be sure that the survey has captured a representative sample of faculty members working in private higher education institutes in Kuwait. Finally, investigating academics’ perceptions provides only one side of the story. What faculty members believe to be true are based on their own experience and assumptions. Full interpretation of the findings requires consideration of the views of students.

## Data Availability

The data that support the findings of this study are available on request from the corresponding author, DE. The data are not publicly available as they contain information that could compromise the privacy of research participants.
